# Differences in attitudes to feeding post repair of Gastroschisis and development of a standardized feeding protocol

**DOI:** 10.1186/s12887-019-1858-z

**Published:** 2019-12-04

**Authors:** Donna Hobson, Kaye Spence, Amit Trivedi, Gordon Thomas

**Affiliations:** 1 Grace Centre for Newborn Care, The Children’s Hospital at Westmead, Locked Bag 4001, Westmead, NSW 2145 Australia; 2 Western Sydney University, School of Nursing and Midwifery, Locked Bag 1797, Penrith, NSW 2751 Australia; 3 The Children’s Hospital at Westmead Clinical School, The University of Sydney, Locked Bag 4001, Westmead, NSW 2145 Australia; 4 Department of Surgery, The Children’s Hospital at Westmead, Locked Bag 4001, Westmead, NSW 2145 Australia

**Keywords:** Gastroschisis, Infant, Newborn, Enteral, Feed, Protocol

## Abstract

**Background:**

The purpose of this study was to examine differences in attitudes to feeding in neonates with Gastroschisis between clinical groups and to develop a standardized feeding protocol. Confusion, inconsistencies in practice and lack of evidence could be contributing to avoidable delays in the establishment of enteral feeds resulting in lengthy requirements for central venous access, dependence on total parenteral nutrition (TPN), increased risk of sepsis, TPN related cholestasis and prolongation in length of hospital stay.

**Methods:**

A national survey of clinicians (neonatologists, neonatal intensive care nurses and paediatric surgeons), looking after neonates with gastroschisis was undertaken to determine differences in feeding practice post repair. In addition, an audit of practice in one hospital was undertaken to examine variations in practices between clinicians. A feeding protocol was then developed using inputs from surgeons and neonatologists.

**Results:**

Gastric aspirates and residuals were typically used as indicators of feed readiness and feed tolerance; however, there was very little consistency within and between clinical groups in definitions of tolerance or intolerance of feeds and in how to initiate and progress feeds. A feeding protocol with clear definition of feed readiness and a clear pathway to progression of feeds was developed to help overcome these variations in practice with the possibility that this might reduce the length of stay (LOS) and have other secondary benefits. The protocol included early introduction of enteral feeds particularly direct breast or sucking feeds.

**Conclusions:**

Wide differences in attitudes to feeding neonates post Gastroschsis repair exist and the need for a consistent protocolized approach was felt. The feeding protocol we developed requires a change of practice and further clinical trials are needed to evaluate its effectiveness.

## Background

Gastroschisis is a congenital abdominal wall defect with an increasing incidence which is easily diagnosed in the antenatal period. It is thought to be the result of an ischemic event in utero involving bowel and other abdominal contents herniating through a weakness in the para-umbilical region [1]. It is postulated that prolonged contact of exposed bowel with amniotic fluid causes inflammatory changes damaging unprotected bowel resulting in the bowel becoming thickened and dilated. This exposure potentially producing an array of complications including bowel matting, volvulus, intestinal atresia and stenosis [2].

Gastroschisis occurs in 5 of 10,000 live births with a survival of greater than 90% however, there is often significant associated morbidity [3, 4]. Difficulties in commencing and progressing enteral feeds [5, 6], is well recognized in this group and this difficulty is thought to be due to the poor gut motility as a result of the bowel exposure and thickening. The delay in the establishment of enteral feeds often contributes to lengthy requirements for central venous access, dependence on total parenteral nutrition (TPN), small bowel bacterial overgrowth (SBBO), increased risk of sepsis and TPN related cholestasis. This can significantly prolong length of hospital stay [7, 8].

As there are 3 clinical groups making decisions on feeding, (neonatologists, neonatal nnurses and paediatric surgeons), differences in approach to feeding creates confusion and potentially conflict in the management of these infants. In addition, this perpetuates a culture of caution and delays in enteral feeding resulting in avoidable use of TPN and all the other issues associated with it. We could not find guidelines or protocols in the literature that addressed this or provided a pathway for feeding.

For these reasons our aims were to examine differences in attitudes to feeding in neonates with Gastroschisis between clinical groups in Australia and to develop a standardized feeding protocol. The protocol would provide surgeons, neonatologists and neonatal nurses with a standardized feeding pathway with several secondary benefits such as earlier suck feeds and potentially an earlier discharge on breastfeeds, with the ultimate aim of improved feeding enhancing weight gain and improving infant physical and mental developmental outcomes.

## Methods

The research was undertaken in three parts; (i) a retrospective audit, (ii) a national on-line survey, and (iii) development of a feeding protocol. These three methods were utilised to ensure all the issues that could potentially lead to inconsistencies in feeding these infants were captured.

### Retrospective unit audit

The retrospective audit was undertaken over a two-year period (2015–2016) of all infants (n-15) admitted to this surgical neonatal intensive care unit (sNICU) with Gastroschisis. The aim was to identify feeding patterns in two groups of infants, those with a primary repair and those who had a staged repair using a silo. At our institution, primary repair is performed only on neonates with uncomplicated Gastroschisis. Decision to pursue primary repair vs delayed staged repair using a silo was individualized by the surgeon looking after the neonate on that day. The unit-based clinical database was reviewed and data of multiple variables were collected. Information obtained from the audit was used to inform the questionnaire regarding variations in practice between the different disciplines of surgeons, neonatologists and neonatal nurses.

### National on line survey

A questionnaire was developed based on literature specifically on Gastroschisis and feeding practices [1, 2, 7–9] (Additional file 1). The questionnaire sought opinion on individual clinician’s practice concerning gastric aspirate volumes, time when feeds are commenced, replacement of gastric losses and gastric residuals once feeds had commenced, methods for progressing feeds, milk types, criteria for stopping feeds and methods of feeding such as tube or suck.

The survey was distributed online using Survey Monkey, SurveyMonkey Inc. San Mateo, California, USA to paediatric surgeons, neonatologists and experienced neonatal nurses working in sNICU across Australia and New Zealand. The distribution list was obtained from the Australia and New Zealand Neonatal Network database and a database of paediatric surgeons in Australia held by the this institution’s Department of Surgery.

### Development of feeding protocol

The protocol was developed by the neonatal nursing team with inputs and assistance from the neonatologists and paediatric surgeons. Survey analysis and literature search provided inputs into ill–understood issues such as gastric residuals, the safety of early enteric or trophic feeds, safe feed volumes, the progression of feeds and the safety of direct breastfeeds. Although there was very little gastroschisis specific literature on feeding, guidelines from other neonatal conditions such as necrotizing enterocolitis (NEC) helped in the development of the protocol. We adopted an approach that intentionally introduced feeds early and encouraged early direct breast or suck feeds. The protocol was constructed in four stages and two parts to enable some flexibility for slow feed tolerance without repeatedly discontinuing feeds. Specific items such as the use of gastric residuals to halt feeds, the timing of commencement of trophic feeds, recommended volumes and staged increases in volume, transition from tube to suck feeds and support for full breastfeeds were used in the design of the protocol.

**Fig. 1:**
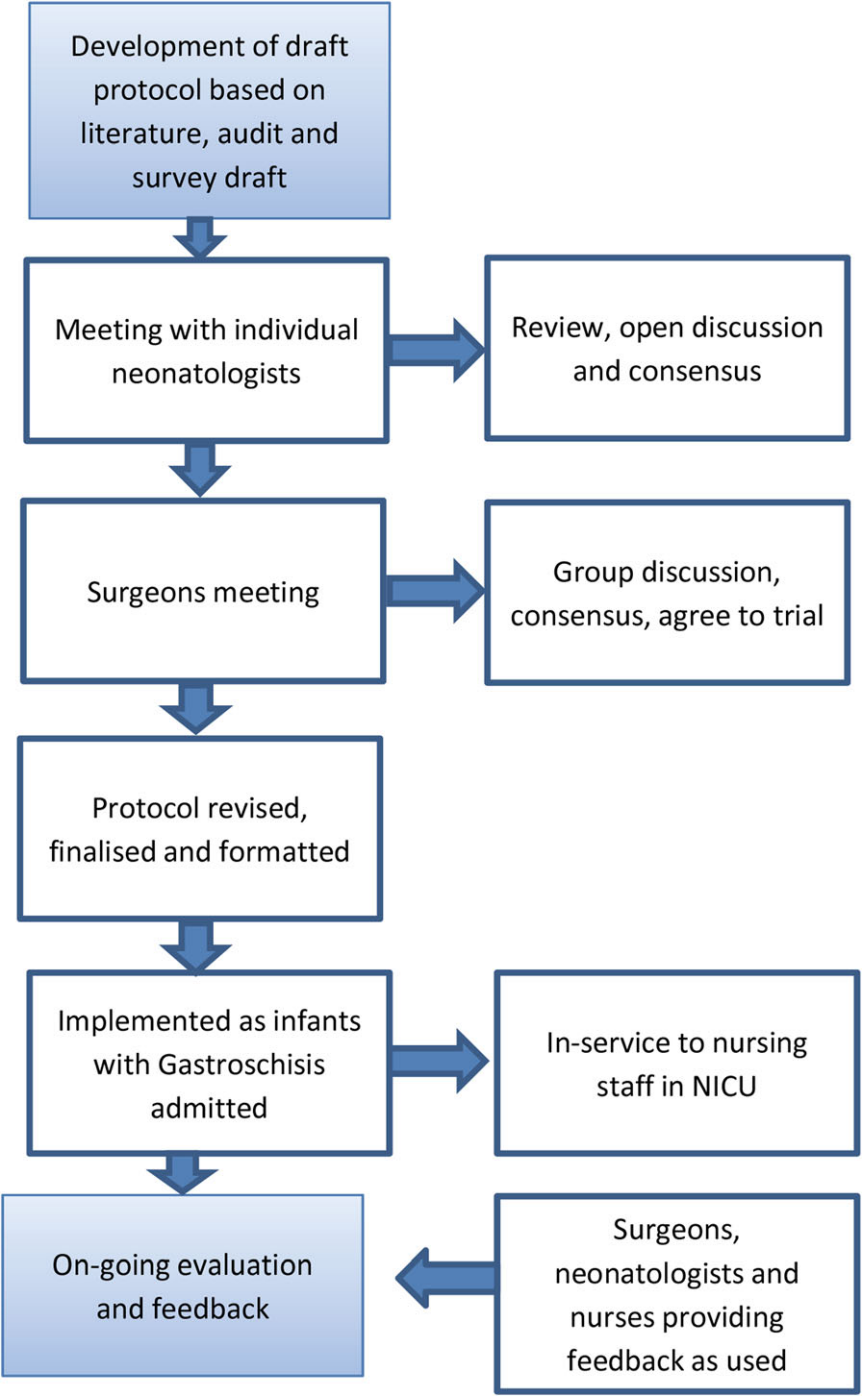
(Colour and Mono) Consensus process for development of feeding protocol

The protocol was sent to the neontologists and paediatric surgeons for comment and then presented at their group meeting. Consensus was obtained before it was implemented (Fig. 1).

The identified outcomes to measure the potential success of the protocol were duration of TPN, length of central venous line (CVL) use, the time to initiate enteral feeds and time to establishment of full breast or enteral feeds. These were divided into primary and secondary outcomes (Tables 1 and 2).

**Table 1:** Positive outcome measures for pathway

Stages of Pathway	Primary Outcome	Secondary Outcome
Stage 1	Trophic feeds commenced 24 post closure of defect	Trophic feeds offered at breast
Stage 2	Suck feeds commenced	TPN reduced
Stage 3	Breastfeeding commenced	TPN ceased CVL removed
Stage 4	Demand breastfeeds Breastfeeding at discharge	Length of Hospital Stay

**Table 2:** Potential Negative Outcomes

Primary	Secondary
Dependence on TPN	TPN related Cholestasis
Sepsis	Small bowel bacterial overgrowth
Prolonged hospital stay	

Statistical analyses of non-parametric tests for comparisons were performed using IBM SPSS, version 24.0 (IBM Corp., Armonk, NY, USA).

## Results

### National online survey

One hundred and ninety-nine on-line questionnaires were distributed in 2016 across Australia and New Zealand. Eighty-one were returned. Twenty surgeons 20 (25%), 33 neonatologists (41%) and 28 experienced neonatal nurses (35%) responded giving an overall response rate of 31%. Eighty-six percent of the respondents had worked with neonates for more than 5 years and 46% more than 10 years. Fifty-seven percent had looked after more than 5 infants with Gastroschisis in the past 12 months.

In the management of feeding of infants with Gastroschisis, current evidence and research (44%), tradition (30%) and personal opinion (12%) influenced the practice. Gastric aspirates were identified by 81% of respondents as an essential part of the management of Gastroschisis. When asked about the management of the volumes of gastric residuals the responses were varied with 44% stating aspirates should be discarded, 23% stating that only part of the aspirate should be returned and 9% stating that all of the aspirate should be returned. The volume returned varied among the practitioners. When examined by professional disciplines there was inconsistencies between the groups with neonatal nurses being more conservative in the volumes returned (Table 3). There were differences between the professional disciplines on the role of sucking to promote gastric motility with 79% of nurses and 30% of surgeons, indicating it did.

**Table 3:** Volume of gastric aspirates returned when on nil enteral feeds – preference by discipline

	Between 1-2mls/kg		Between 3 and 4 ml/kg		Between 5 and 6 ml/kg		Return the entire aspirate		Discard the aspirate	
	N	%	N	%	N	%	N	%	N	%
Surgeon (20)	2	10	8	40	1	5	2	10	7	35
Neonatologist (33)	6	18	12	36	0	0	5	15	10	30
Neonatal Nurse (28)	1	25	0	0	2	7	7	25	18	64

The opinions varied when it came to commencing trophic feeds. Once again there was variation between the professional disciplines (Table 4).

**Table 4:** Commencement of Trophic Feeds – preference by discipline

	Within 4 h silo/repair		First post-op day		Bowel sounds present		Bowels opened		When aspirates clear	
	N	%	N	%	N	%	N	%	N	%
Surgeon (20)	3	15	1	5	4	20	2	10	5	4
Neonatologist (33)	4	12	8	24	5	15	2	6	9	27
Neonatal Nurse (28)	2	7	1	4	3	11	3	11	14	50

**Fig. 2:**
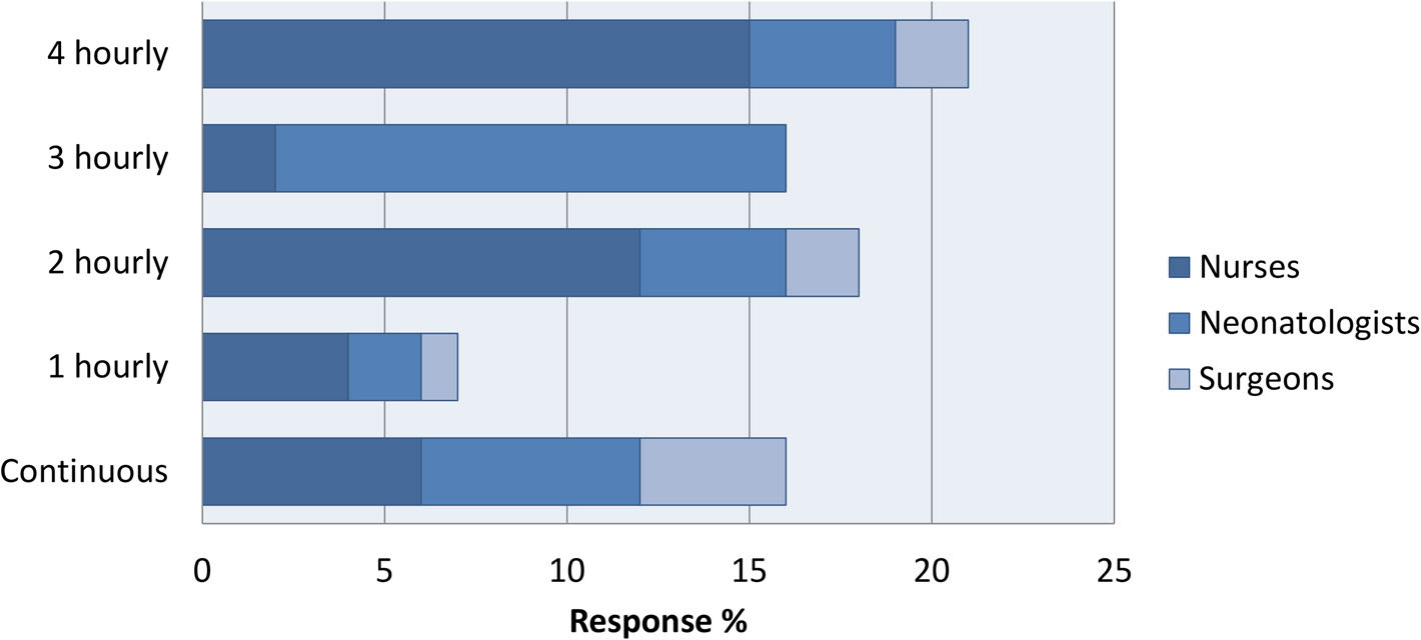
(Colour and Mono) Preferred frequency for commencing feeds by discipline

**Fig. 3:**
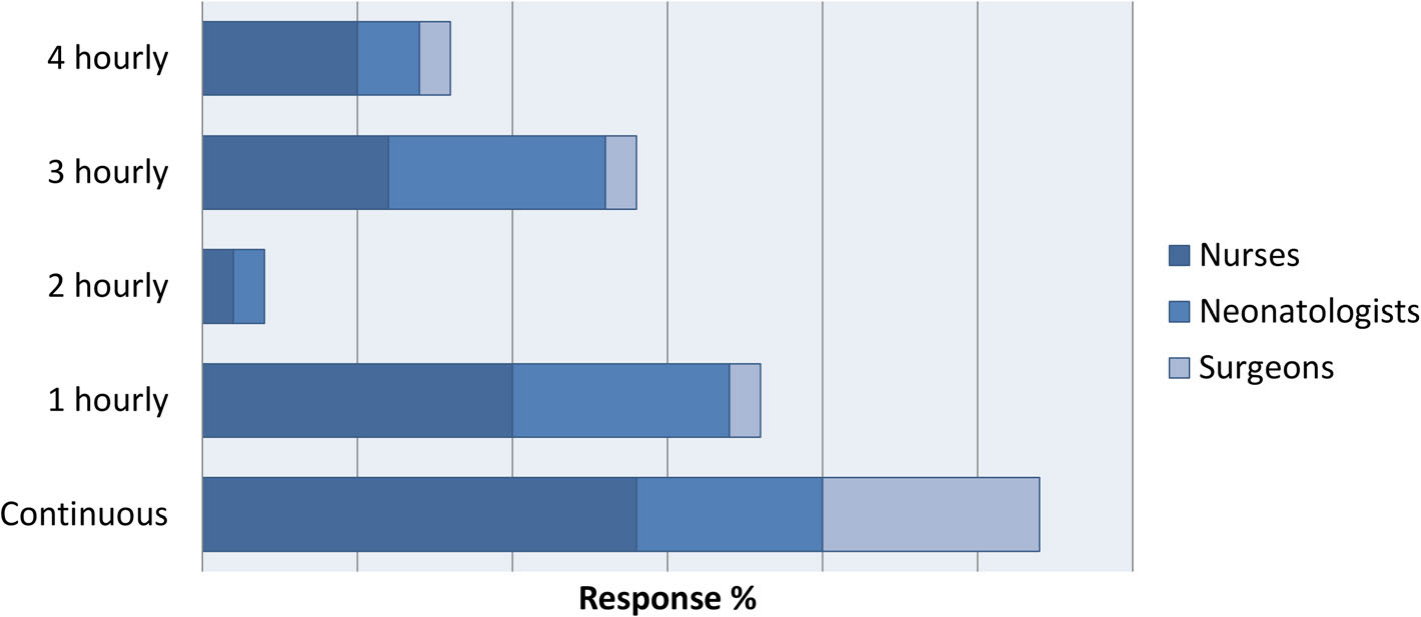
(Colour and Mono) Preferred frequency of grading up feeds by discipline

**Fig. 4:**
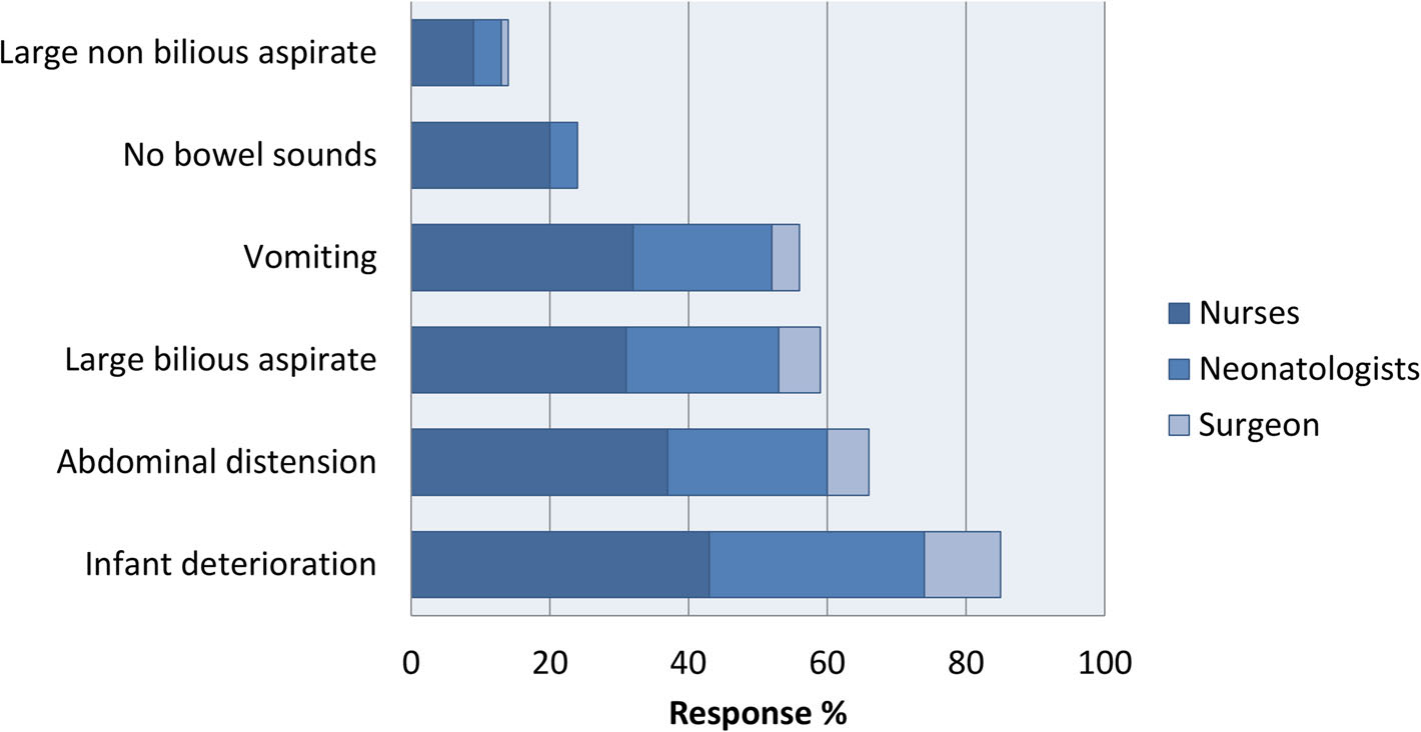
(Colour and Mono) Reasons for ceasing feeds by discipline

The opinions also differed in the preferred methods of delivery of feeding, grading up and reasons for cessation of feeding (Figs. 2, 3, 4). When asked if a structured feeding plan for the management of feeding infants with Gastroschisis may prevent unnecessary stop/start of feeds, 82% said yes.

### Audit of practice in one unit

A total of 15 infants with simple gastroschisis were included in the retrospective audit. Ten (66.6%) had a primary repair and five (44.4%) had a staged repair with a silo. Table 5 shows the demographic details and differences in feeding milestones between the groups. Gestational age and birth weight were similar for both groups. Median LOS for infants with a primary repair was shorter compared infants with a staged repair. Infants who had a staged repair took on average 10 days longer to commence feeds post-operatively, longer for their first suck feed, to achieve full enteral feeds and longer to achieve full suck feeds. Once enteral feeds were commenced the time taken to establish full enteral feeds were similar in both groups.

**Table 5:** Comparison between closure type for Gastroschisis

Type of Closure		GA (weeks)	BW (gm)	Length of Stay (Days)	TPN (Days)	CVL (Days)	Start feeds post-op (Days to)	Achieve full feeds (Days)	First suck feed post-op (Days to)	Achieve full suck feeds (Days to)
Primary repair	Median	36	2355	20.05	18	18	3.5	17	15	9
	N	10	10	10	10	10	10	10	9*	9*
	Std. Deviation	1.687	635.04	10.83	9.38	8.87	1.75	8.46	5.38	7.67
Staged Silo	Median	36	2300	54	31	28	13	16	23	14
	N	5	5	5	5	5	5	5	5	5
	Std. Deviation	1.82	704.19	62.43	35.5	15.16	12.63	13.36	13.16	9.12

^*^One infant returned to birth hospital before suck feeds established

The audit found there were frequent changes made in feeding regimes that led to feeds being ceased or delayed due to concerns about feed tolerance. These concerns were often due to measuring aspirates and residuals which were inconsistent between staff within the shift or over a period of days.

### Development of a Gastroschisis feeding pathway for implementation in a surgical NICU

There was uniform consensus between all groups that the protocol was needed and could help. Concerns were raised as there was a lack of literature and evidence to support a protocolized approach to feeding. The intentional early introduction of enteral feeds especially direct breast feeds was debated in an open forum that included all three clinical groups at one of their scheduled meetings. Some of the surgeons felt early adoption of oral feeds was important in facilitating faster recovery and gut motility A consensus was reached and the decision to implement the pathway was made. There was potential bias in this process as the discussion and eventual agreement was held in an open forum.

**Fig. 5:**
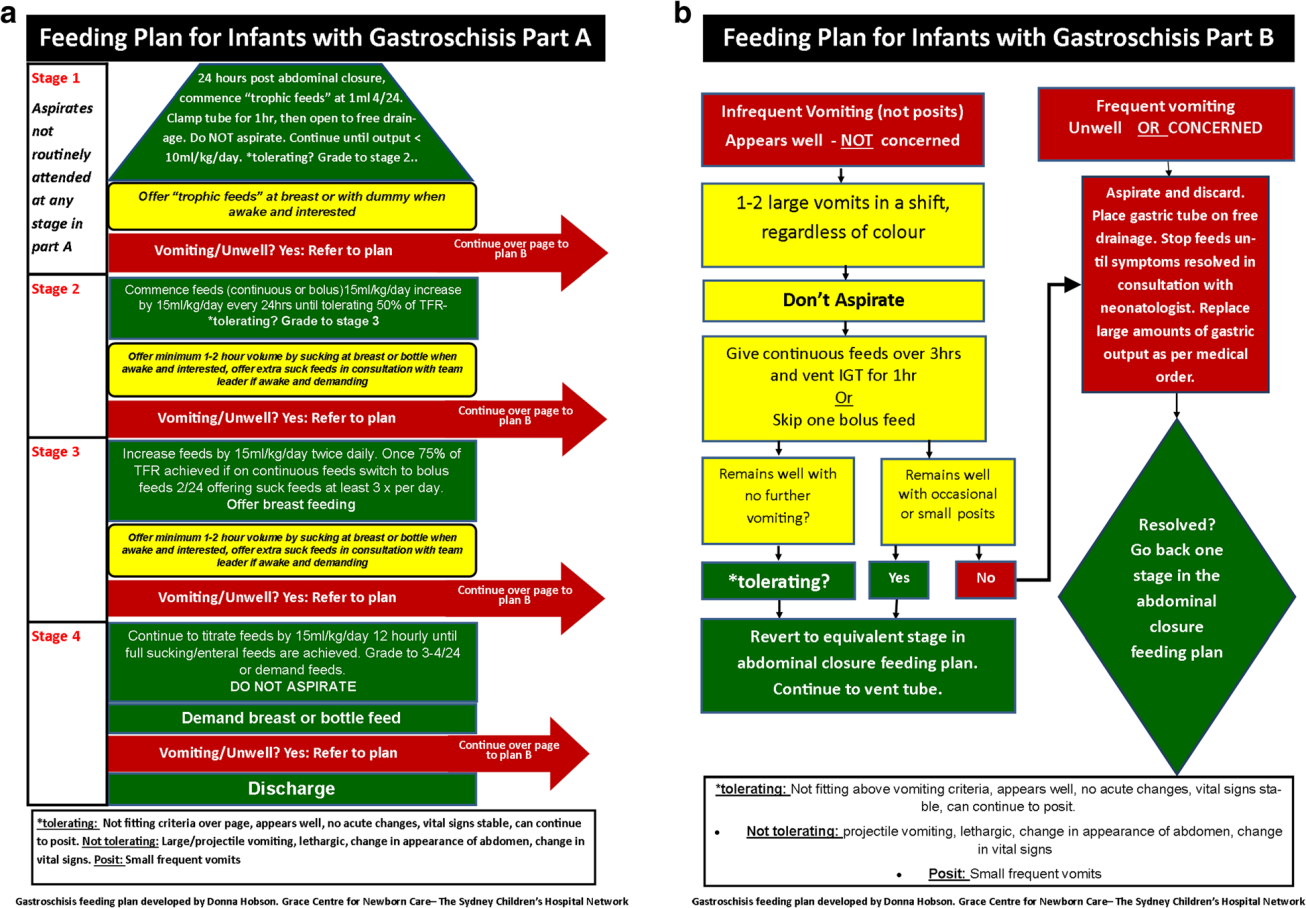
**a** and **b** (Colour) Gastroschisis Feeding Protocol

Feeds were to be commenced 24 h after abdominal wall closure providing the infant was breathing spontaneously. It was agreed that use of the pathway for an infant was at the discretion of the consulting surgeon and neonatologist and at this point should only be used for infants with uncomplicated Gastroschisis (Fig. 5). The pathway was designed in four stages of progression and measurable outcomes identified for each stage. These outcome measures were established to measure the success of implementing the protocol and these are shown in Table 1.

In addition negative outcomes were identified to enable a thorough evaluation of the protocol.

## Discussion

By undertaking an audit, we were able to identify inconsistencies in the local environment that led to the delay in the introduction of enteral feeds and the lack of sucking feeds being offered. Lack of clarity particularly about aspirates and residuals led to frequent changes in feeding regimes between nursing shifts. The survey of various clinicians across the country supported the inconsistencies found in one hospital.

This innovative feeding protocol that we developed for infants with Gastroschisis enables a standardized approach that bedside nurses looking after babies with Gastroschisis can follow. We believe this will facilitate faster progression to full sucking feeds and possibly earlier discharge. Previous reports that have used protocols have shown similar benefits but have been applied retrospectively to data cohorts and have been less specific [10]. Bulter et al. [11] demonstrated that a standardized feeding guideline within a surgical NICU for very low birth weight (VLBW) infants improved outcomes and reduced costs. It included enteral nutrition being initiated by 24 h of age and then advanced by following the feeding guidelines [11]. This guideline continued to rely on aspirates and residuals however a pathway was developed to guide their management and defined ‘normal’ and ‘not normal’ when measuring aspirates and residuals. While this study does not specifically discuss infants with Gastroschisis, it does address the inconsistencies of feeding practices within the surgical NICU. The implementation of the guideline stopped the variation in feeding practices improved feeding outcomes without any increase in complications for example, NEC, sepsis, line infections or mortality.

Our feeding protocol introduced a change in practice as it introduced tropic feeds earlier and supported advancement of feeds without focussing on gastric residuals. Moreover, intentional early introduction of sucking feeds was important in facilitation faster discharge [12–14]. A consensus approach that involved inputs from both surgeons and neonatologists helped ensure compliance.

The protocol (Fig. 5) is in two parts (A and B) which enables the clinicians to choose to deviate if there is vomiting or excessive gastric residuals. Once settled, the feeding regime continues where left off in Part A. This ensures progression of feeds rather than the regression that occurred previously. The protocol is currently being used in practice and is being evaluated against the outcome measures listed previously.

There were a few limitations of this study that need to be highlighted. The consensus process of open forum has the potential for bias due to possible coercion from individuals. The audit was only at a single site in the author’s neonatal unit. To evaluate its’ effectiveness, on-going research and testing of the protocol through undertaking a prospective observational study tracking success of implementation and measuring on-going outcomes would be an appropriate plan.

## Conclusions

Wide differences in attitudes to feeding neonates post Gastroschsis repair exist and the need for a consistent protocolized approach was felt. The feeding protocol we developed requires a change of practice by introducing trophic feeds earlier and supports advancement of feeds without focussing on the gastric residuals and provides a pathway nurses, surgeons and neonatologists can use that standardizes how feeds are initiated and progressed with potentially beneficial results. Further studies are needed to evaluate its effectiveness.

## Supplementary information

**Additional file 1.** Gastroschisis and Feeding Questionnaire.

## Data Availability

The datasets used and/or analysed during the current study are available from the corresponding author on reasonable request.

## Abbreviations

CVL: Central venous line
LOS: Length of stay
NEC: Necrotising enterocolitis
SBBO: Small bowel bacterial overgrowth
sNICU: Surgical neonatal intensive care unit
TPN: Total parenteral nutrition
VLBW: Very low birth weight
