# Supplementary material for: Differences in attitudes to feeding post repair of Gastroschisis and development of a standardized feeding protocol
Source: BMC Pediatr. 2019 Dec 4;19:475. doi: 10.1186/s12887-019-1858-z (PMC6894280; doi:10.1186/s12887-019-1858-z)
Supplement: Supplementary file 1 — Additional file 1. Gastroschisis and Feeding Questionnaire. [file 12887_2019_1858_MOESM1_ESM.pdf]

Gastroschisis and Feeding Questionnaire.

What influences your practice in the management of infants with Gastroschisis?

a) Traditional management practices
b) Current evidence and research
c) Personal opinion and experience
d) Other
e) Comments

The following questions are on gastric aspirates when <u>NIL BY MOUTH</u> infant:

Do you believe measuring the volume of gastric aspirates is an essential part of gastroschisis management?

Yes □ *No* □ *Not Sure* □

Please comment

Do you believe gastric aspirates are affected by the position of the infant at the time of measurement i.e. side lying, prone or supine?

Yes □ *No* □

Please comment

While an infant is *<u>nil by mouth</u>*, how often would you measure gastric aspirates?

a) Hourly
b) Second hourly
c) 3 – 4 hourly
d) 6 – 8 hourly
e) Other

Please comment

Does the colour/composition of the gastric aspirate (i.e. dark green, blood stained) influence your decision to return or discard a gastric aspirate?

Yes □ *No* □

Please comment

While an infant is nil by mouth do you feel that a gastric aspirate should be:

a) Discarded
b) All of the aspirate should be returned
c) Part of the aspirate should be returned
d) Other

Please comment

If returning a gastric aspirate for an infant that is *<u>nil by mouth</u>* do you have a preference on the amount that should be returned?

a) Between 1-2mls/kg
b) Between 3-4ml/kg
c) Between 5-6ml/kg
d) Return the entire aspirate
e) I would discard the aspirate

Please comment

When would you replace electrolytes intravenously?

a) When gastric aspirates are >10ml/kg/day

b) When gastric aspirates are >20ml/kg/day
c) Straight away
d) After checking the aspirate electrolytes
e) When serum electrolytes are deranged
f) Other

Please comment

To correct or maintain electrolytes in an infant with large gastric aspirates/large vomits you prefer to:

a) Increase the TFR (Total Fluid Requirement, generally TPN)
b) Add a sideline of required fluid/electrolytes
c) Other

Please comment

The following questions relate to <u>FEEDING</u> infants with gastroschisis:

Who do you believe is the ultimate decision maker in commencing and grading up of feeds?

a) Consultant Surgeon
b) Surgical Fellow/Registrar
c) Consultant Neonatologist
d) Neonatal Fellow/Registrar
e) Nurse Practitioner
f) Nurse Unit Manager
g) Joint Decision

Please comment

When would you commence trophic enteral feeds?

a) Within 4 hours after silo insertion/primary repair
b) The first post-operative day
c) Once bowel sounds can be auscultated
d) When the aspirates are clear and minimal
e) Not until the bowels have been opened
f) Other

Please comment

What do you believe is the best method of *<u>commencing</u>* feeds?

a) Continuous
b) Every hour
c) Every second hour
d) Third hourly
e) Every four hours
f) Other

Please comment

What do you believe is the best method of *<u>grading up</u>* feeds?

a) Continuous
b) Every hour
c) Every second hour
d) Third hourly
e) Every four hours
f) Other

Please comment

If breast milk is unavailable do you have a preference for a type of formula to be used?

Yes □ *No* □

If Yes, would you choose

a) Standard
b) Hydrolysed (e.g. Neocate)
c) Semi Hydrolysed (e.g. Pepti Junior)
d) Other, please specify

Please comment

Do you think it is important to regularly check gastric residuals in infants with gastroschisis once feeding has commenced?

Always□ *Sometimes* *No* □

Please specify/comment

If returning a gastric residual *__(Infant on milk feeds)__* do you have a preference on the amount of residual that should be returned?

a) Between 1-2mls/kg
b) Between 3-4ml/kg
c) Between 5-6ml/kg
d) Return the entire residual
e) I would discard the residual

Please comment

Your criteria for stopping feeds would be *(Please select all that apply):*

a) Infant deterioration (febrile, sepsis etc.)
b) Abdominal distension, appearance, firmness
c) Vomiting
d) Large bilious aspirates >10ml/kg/day
e) Unable to auscultate bowel sounds
f) Large non-bilious aspirates
g) Other

Please comment

Do you believe that correct milk temperature (body temperature) encourages gastric motility?

Yes □ *No* □

Please comment

Do you believe offering oral sucking feeds encourages gastric motility?

Yes □ *No* □

Please comment

General Questions

Do you believe the addition of an oral sodium supplement would assist with absorption of feeds in the small intestine and increase feed tolerance)?

Yes □ *No* □

Please comment

In your opinion would pro kinetic medications assist with improving gut motility in infants with gastroschisis?

Yes □ No □

Please comment

Do you believe suppositories should be used for infants with Gastroschisis?

Yes □ No □

Please comment

Do you think probiotics could be used as a possible adjunct treatment in infants with gastroschisis?

Yes □ No □

Please comment

Do you think a predictive bowel scoring method would assist in developing a multidisciplinary plan for infants with gastroschisis? *(Gastroschisis prognostic score www.capsnetwork.com.au) (Cowan, Puilgandla, Laberge, et. al, 2012)*

Yes □ No □

Please comment

Do you believe a structured feeding plan for the management of feeding infants with Gastroschisis may prevent unnecessary stop/start of feeds?

Yes □ No □

Please comment

Is there anything else you would like to suggest that you think would improve the management of infants with gastroschisis?

Please comment

Now for some questions about you:

Please indicate your position

a) Surgeon (Consultant, Fellow, Registrar)
b) Neonatologist (Consultant, Fellow, Registrar)
c) Nursing unit manager/Nursing Team Leader
d) Clinical nurse specialist/Neonatal nurse practitioner
e) Registered nurse

How long have you worked in your current position?

a) More than 10 years
b) Between 5 and 10 years
c) Between 2 and 4 years
d) Between 1 and 2 years
e) Between 6 months and 1 year
f) Less than 6 months

How many gastroschisis infants have you cared for in the past 12 months?

a) 10 - 12
b) 5 – 9
c) 1 – 4
d) None

Thank you for participating.
